# Cardiovascular and Autonomic Responses to Energy Drinks—Clinical Implications

**DOI:** 10.3390/jcm9020431

**Published:** 2020-02-05

**Authors:** Kiran R. Somers, Anna Svatikova

**Affiliations:** 1 Department of Cardiovascular Diseases, Mayo Clinic, Rochester, MN 55905, USA; kiran.somers@gmail.com; 2 Department of Cardiovascular Diseases, Mayo Clinic, Scottsdale, AZ 85259, USA

**Keywords:** autonomic function, sympathetic activity, catecholamines, blood pressure, energy drinks, caffeine

## Abstract

There is an increasing consumption of energy drinks both in the United States and worldwide. The components of these beverages are sometimes unclear but commonly include caffeine, sugars, taurine, and B-vitamins. Young people, particularly those engaged in sports, studying, and in the military are especially likely to be consumers of energy drinks. While limited data are available regarding their autonomic and hemodynamic effects, current literature suggests that energy drink consumption is accompanied by increases in blood pressure, sympathetic drive, and also in QT prolongation. There are no systematic long term studies identifying consequences of frequent energy drink consumption. However, multiple anecdotal reports implicate energy drinks in adverse cardiovascular events including atrial fibrillation, ventricular arrhythmia, myocardial infarction, and sudden death. Events such as atrial fibrillation may even occur in otherwise healthy subjects with structurally normal hearts. It is likely that these cardiovascular outcomes are triggered by the hemodynamic, autonomic, and electrocardiographic responses to energy drink consumption. What remains unclear is how concomitant use of other stimulants such as amphetamines and nicotine may interact to potentiate neural and circulatory responses and cardiovascular consequences when combined with energy drinks.

## 1. Introduction

Energy drinks were first introduced in the United States more than three decades ago. Since then, national consumption has been rapidly increasing, with particularly strong penetration and regular use among young adults, amateur athletes, and military personnel. These beverages are marketed on the premise of increasing energy, mental focus, wakefulness, and fatigue prevention. However, the burgeoning usage of energy drinks has been associated with an increased risk of serious health consequences. This review examines the composition of energy drinks, their effects on cardiovascular hemodynamics, and potential mechanisms by which energy drinks increase cardiovascular risk. Particular focus is on exploring the neural and circulatory responses to energy drink intake.

## 2. Consumption of Energy Drinks

In the United States, 51% of college students consume at least one energy drink per month [1] and almost a third of students between grades 8 and 12 drink them [2]. Importantly, 45% of deployed military personnel are reported to consume a minimum of one energy drink daily, with 14% having three or more [3]. While this increase can be attributed to several factors, most notable are the aggressive marketing, increased distribution, and a growing consumer base. According to Allied Market Research, the global energy drink market was valued at $53 billion dollars in 2018 and will increase to $86 billion by the year 2026 [4,5].

Although the market is growing rapidly, the adverse effects of energy drink consumption raise concerns. Energy drink advertising has been a particular target for criticism due to the marketing of the beverages to minors [6]. This is a group with developing neural and cardiovascular systems and the consequences of energy drink use are unclear and unpredictable.

## 3. Composition of Energy Drinks

Energy drinks contain variable combinations of caffeine, sugar, amino acids like taurine, guarana, glucuronolactone, herbal extracts (ginseng, gingko biloba, milk thistle), vitamins (especially high doses of B vitamins, including B12), and other additives [7,8]. The contents of several widely available energy drinks are listed in Table 1. They typically contain between 70 to 200 mg of caffeine per 16 fl oz [7]. For reference, an 8 oz cup of brewed coffee contains 95 mg of caffeine per cup, and a 1 oz shot of espresso contains 63 mg of caffeine [9]. Notably, a single energy drink may be up to 32 fl oz, implying up to 400 mg of caffeine, the equivalent of more than six 1 oz shots of espresso.

The composition of energy drinks also needs to be considered in a context in which these beverages are increasingly being used in combination with alcohol, nicotine, and other stimulants, especially by adolescents and young adults [2]. For example, the consumption of energy drinks in combination with alcohol is a significant public health concern; it is especially prevalent in underage drinkers, is associated with binge drinking and impaired driving, and is considered riskier than consuming alcohol alone [10].

## 4. Effects of Caffeine

Caffeine is a widely consumed phytochemical present in beverages, food, and over-the-counter medications. Caffeine is considered a drug and works as a central nervous system stimulant [11]. It is a lipophilic molecule which easily crosses the blood–brain barrier, and increases neurotransmitter concentration in the brain [11,12]. Caffeine’s greatest effect takes place in the basal ganglia, where its inhibitory action on adenosine receptors and synergistic effect with dopamine turn off pathways which act to restrict motor activation signals in the brain [12].

Caffeine is structurally similar to adenosine and acts as a competitive inhibitor for adenosine receptors. Caffeine binds to the receptor in place of adenosine, thus inactivating the inhibitory effect of adenosine binding on dopamine release, leading to further increases in dopamine and greater arousal [12].

Caffeine is also notable for its ability to delay the onset of fatigue and decrease perceived exertion through increased release of excitatory neurotransmitters in the central nervous system [13]. These effects may help explain why caffeine-containing preparations are heavily used in sports and by students. It has been shown to delay fatigue at exercise at 80%–85% VO2 max, increase endurance and performance in aerobic exercise bouts lasting 30 min to an hour, and significantly increase athletic performance in timed trials of 2000 m rowing and 1500 m swimming [11]. Caffeine’s enhancement of cognitive performance in states of low arousal and sleep deprivation are well documented [14]. In a randomized study of three different caffeine preparations (5-Hour Energy, Starbucks DoubleShot, and 3 mg/kg of caffeine powder) versus placebo, Paulus et al. reported that all caffeine groups showed elevations in mood, faster reaction times, and improved cognition [15]. Importantly, doses of more than 200 mg caffeine are often associated with side effects such as insomnia, headaches, tachycardia, and arrhythmia [16,17].

Regarding the effects of caffeine on heart rate at rest and exercise, Nishijima et al. reported no changes in resting heart rate [18], while Yeragani et al. noted an increase in both heart rate and in the high-frequency component of heart rate variability [19]. During exercise, the Nishijima et al. study observed an increase in the low-frequency component of heart rate variability while Yeragani et al. noted a reduction in the high-frequency power [18,19]. These findings during exercise are consistent with reciprocal increases in sympathetic tone and decreases in vagal drive during exercise. From a clinical perspective, insofar as the effects of caffeine can be inferred from studies of coffee intake, coffee has been implicated as a trigger for acute myocardial infarction [20,21]. In a study of more than 500 incident cases of non-fatal myocardial infarction, Baylin et al. found that within an hour after coffee consumption, the relative risk of myocardial infarction was 1.49. This association was especially marked in people who drank one or fewer cups of coffee per day, those with sedentary lifestyles, or those with three or more risk factors for heart disease [21]. The apparent heightened risk of acute myocardial infarction in non-habitual coffee drinkers is complemented by studies of the long term effects of habitual coffee consumption. In a critical review of the relationship between caffeine and cardiovascular diseases, Zulli et al. addressed relevant potential mechanisms and noted reduced risks of both cardiovascular and all-cause mortality in regular coffee drinkers. The arrhythmogenicity of caffeine appears to be dose-dependent, since typical levels of caffeine/coffee consumption had no effect in terms of any increased risk of atrial fibrillation and ventricular arrhythmias [22].

## 5. Effects of Sugar

Not all energy drinks contain sugars; however, those that do contain variable combinations of glucose, sucrose, fructose, or high-fructose corn syrup. Fructose appears to have the greatest autonomic effect, significantly increasing blood pressure (BP) over a 2 h period after consumption in healthy young humans. BP increased rapidly 30 min after fructose consumption and systolic BP peaked at 6.2 ± 0.8 mmHg higher than baseline [23]. In children between the ages of 11 and 12, consumption of sugary beverages caused a small but significant increase of 0.8 mmHg in systolic BP, likely due to sympathetic nervous system activation [24].

We have previously published a randomized double-blind study investigating the cardiovascular responses to Rockstar energy drink versus placebo in healthy adults. The placebo drink contained the same level of carbohydrate as the energy drink. Placebo increased plasma glucose by more than 50 mg/dL, systolic BP by 3 mmHg, heart rate by 7 beats/min and plasma norepinephrine by more than 30% [25], suggesting a potent sympathetic excitatory effect of sugar alone.

## 6. Effects of Other Energy Drink Ingredients

While caffeine can improve sports performance, other ingredients commonly found in energy drinks, such as guarana and ginseng, can also attract athletes to these legal performance-enhancing cocktails. Guarana comes from a plant which produces seeds containing small amounts of caffeine, theophylline, and theobromine. Guarana is included in many supplements as an appetite suppressant and to increase sports performance.

Ginseng has been used for centuries as a form of medicine, but more recently has been included as an ergogenic aid. Ginsenocides are believed to play a role in mediating the catabolic effects brought on by cortisol and also in alleviating fatigue [11]. However, further research is required to fully understand ginseng’s mechanistic contributions to enhancing sports performance.

Regarding the effects of energy drinks on BP, the amino acid taurine has been shown to significantly decrease BP in certain populations. A randomized, double-blind, placebo control trial in prehypertensive individuals showed a drop of 7.2 mmHg in mean systolic BP and a 4.7 mmHg drop in diastolic BP with only a 1.6 g per day dosage of taurine over 12 weeks. The same study showed a 3.8 mmHg and 3.5 mmHg drop in mean ambulatory systolic and diastolic BP, respectively [26]. Taurine is found abundantly in meat and seafood, and relatively little is known about its neural and endocrine effects. The available data have been recently and comprehensively reviewed by Caine and Geracioti [27].

## 7. Neural, Circulatory and Electrocardiographic Effects of Energy Drinks

With increasing consumption of energy drinks, several studies have investigated their cardiovascular responses. However, results are inconsistent, which is likely due to variations in the brand of energy drink, the volume consumed, and the duration and intensity of post-consumption monitoring. In a study of the effects of Red Bull, Ragsdale et al. showed no significant increases in BP after intake of energy drinks, neither at rest nor during exercise or cold stress. In fact, Red Bull attenuated the BP response to the cold pressor test. Regarding its neural effects, Red Bull elicited a significant increase in pain tolerance in all subjects [28]. However, in a randomized cross-over study of Red Bull (114 mg of caffeine) versus tap water, Grasser et al. noted a significant increase in systolic BP (5.2 mmHg) and diastolic BP (6.1 mmHg) as well as increases in heart rate of about 3.7 beats/min and cardiac output [29]. Microvascular endothelial function and total peripheral vascular resistance did not change significantly, suggesting that an increase in cardiac chronotropic drive, perhaps by sympathetic activation and/or vagal withdrawal, was the underlying mechanism. In a subsequent randomized cross-over study of Red Bull (114 mg of caffeine) versus tap water, these investigators again noted increases in systolic BP (7 mmHg), diastolic BP (4 mmHg) and heart rate (7 beats/min). Addition of mental stress resulted in further increases in BP and especially in heart rate, with the combination of Red Bull and mental stress increasing heart rate by 20 beats/min [30]. The increase in BP in subjects placed under mental stress that was noted in this study was absent in the study performed by Svatikova et al. using a Rockstar energy drink containing 240 mg of caffeine [25], more than double the caffeine of the previous study [30]. The reasons for the inconsistent effects of mental stressors on BP (as well as other variables) after energy drink consumption may be explained in part by differences in energy drink composition and dosages, timing and types of stressors used, frequency and methods of BP measurement, subject demographics, and other methodologic factors.

In a double-blind, randomized, placebo-controlled study, Nelson et al. explored the effects of Monster energy drink on heart rate and both time- and frequency-domain measures of heart rate variability, often regarded as a measure of cardiac autonomic modulation. The energy drink content was modified to 2 mg caffeine per kg of bodyweight of the subjects. While the energy drink significantly increased resting heart rate to 65 ± 10 bpm versus 58 ± 8 bpm with placebo, no significant effect of energy drinks on time domain, frequency domain, or sample entropy heart rate variability was evident. Interestingly, the energy drink also did not increase exercise capacity, measured as ride time to exhaustion [31].

In an uncontrolled seven-day study of 15 subjects given two 500 mL cans of an energy drink (brand unspecified) containing 100 mg of caffeine per can on the first day, 500 mL per day for the next five days, and two 500 mL cans again on day seven, Steinke et al. noted significant increases in systolic and diastolic BP (between 7% and 10%), significant increases in heart rate of between 7% and 11%, and significant increases in QTc interval of up to 5%—most marked on day seven. Although autonomic effects were not measured directly, the simultaneous increase in both BP and heart rate is strongly suggestive of an increase in central sympathetic outflow to both the heart and the peripheral vasculature, since otherwise the increase in BP would be expected to elicit a slowing of heart rate through baroreflex-mediated cardiac vagal activation [32].

The concept of increased sympathetic outflow mediating an increase in BP after consuming energy drinks is supported by a randomized, double-blind, placebo-controlled study by Svatikova et al. Plasma levels of norepinephrine, heart-rate, and BP were compared in subjects after consuming a 16 fl oz Rockstar energy drink containing 240 mg of caffeine or a placebo drink of similar caloric value. Energy drinks induced more than 6% increases in both systolic and diastolic BP, but no significant difference in heart rate changes between the two groups. Especially striking was the change in norepinephrine levels, which increased by almost 74% after the energy drink versus 31% after placebo. The increase in catecholamine levels speak to significant increases in sympathetic activation with energy drink intake, further supported by the absence of any heart rate slowing despite the increase in BP [25], as noted earlier by Steinke et al. [32].

While most prior studies have examined the effects of a single can of energy drink, a very recent study explored the consequences of high-volume (32 fl oz) energy drink consumption on BP and the QT interval. They compared two different energy drinks versus placebo, and noted that the energy drinks increased both systolic and diastolic BP. Again, consistent with many prior studies, heart rate was unchanged, and did not decrease as would be expected with a reflex response to the BP increase. Importantly, the QTc increased by almost 20 ms, a clinically significant change in terms of risk of arrhythmia [33].

That these effects are not primarily caffeine-mediated is suggested by Fletcher et al., who conducted a randomized, double-blind, cross-over, controlled study examining ECG and hemodynamics after energy drink intake versus a beverage with an equivalent caffeine content [34]. Subjects consumed a drink containing 320 mg of caffeine versus a “high-volume” energy drink containing 320 mg of caffeine. Notably, 22% of the subjects who consumed the energy drinks experienced palpitations, an adverse effect that was not present in the caffeinated beverage group. Furthermore, the group that consumed the energy drink experienced a significantly prolonged QTc interval of 10 ms at 2 h after consuming the energy drink and an elevated peripheral systolic BP of 4 mmHg at 6 h after energy drink consumption [34]. The QT prolongation is clinically meaningful since, as pointed out by Fletcher et al., the Food and Drug Administration requires thorough investigation of QT prolongation effects of all new drugs, with increases of more than 10 ms raising regulatory concern [35]. The clinical significance of QT prolongation is further highlighted by evidence that consumption of at least 16 oz of a highly caffeinated (160 mg caffeine) energy drink was associated with unmasking of congenital type 1 long QT syndrome (LQTS) in a 13-year-old female who presented to the emergency department with palpitations and chest pain [36]. She subsequently underwent genotype confirmation of LQTS. While caffeine alone may not be the singular driving factor behind cardiovascular risks in energy drink consumption, and various additives and proprietary blends may also play a significant role [8], Dufendach et al. reinforce the concern that energy drinks are often marketed to children, for whom there are little data regarding the effects of caffeine [37].

The acute effects of energy drink consumption are not limited to increases in catecholamines, BP and QT interval. In young adults, increases in platelet aggregation and decreased endothelial function have also been observed [38]. The platelet effects are unlikely to be explained by caffeine, since caffeine does not appear to increase platelet aggregation [39,40]. It is likely that sympathetic activation may be implicated, at least in part, since heightened adrenergic drive has been shown to increase platelet activation [41,42]. However, effects on endothelial function may indeed be in part due to the effects of caffeine, which has been reported to attenuate brachial artery flow-mediated dilation [43].

## 8. Cardiovascular Complications of Energy Drinks

The adrenergic, pressor, and electrocardiographic responses to energy drinks are strongly suggestive of potential to elicit ischemic and arrhythmic sequelae, particularly in individuals with an underlying vulnerable substrate. This risk may be potentiated when energy drinks are used in conjunction with other stimulants such as cigarette smoking. However, despite their widespread consumption, there are no systematic prospective studies of clinical cardiovascular outcomes in response to energy drinks. Nevertheless, numerous anecdotal reports have emerged, some of which are listed below.

In a review of anecdotal reports of adverse cardiovascular effects of energy drinks, Goldfarb et al., described the development of new atrial and ventricular arrhythmias, unmasking of both LQTS and Brugada ECGs, acute coronary vasospasm, ST segment elevation myocardial infarction, and sudden death. Many of these patients did not have any identifiable cardiac abnormality [44]. A later report described a 26-year-old male smoker who often consumed 8–10 cans of energy drinks a day, who presented with acute inferior ST elevation myocardial infarction [45]. Three young healthy males who consumed large amounts of energy drinks were also reported to have developed acute and highly symptomatic atrial fibrillation [46]. That these events can occur in otherwise healthy young individuals without underlying cardiovascular disease speaks to the potency of the effects of energy drinks on arrhythmogenesis, particularly when consumed rapidly and in large quantities.

The risk is likely even higher in patients with structurally abnormal hearts. Ward et al. describe a patient with repaired Tetralogy of Fallot experiencing non-sustained VT and eventually, ventricular fibrillation after consuming three Red Bull energy drinks over 3 to 4 h [47].

## 9. Summary and Conclusions

Energy drinks are being consumed with increasing frequency, particularly by young adults. Randomized trials show significant increases in norepinephrine and BP, as well as QTc prolongation. Anecdotal reports of clinical outcomes include unmasking of underlying channelopathies, atrial fibrillation, myocardial infarction, ventricular fibrillation, and sudden death. The composition of these drinks are often not fully known. Therefore, it is not always clear as to which particular component or combination of components may be responsible for any particular clinical presentation. It is also not clear which energy drinks may be most likely to elicit adverse consequences, especially since the published reports of energy drink clinical trials may not actually name the specific drink tested.

There is a compelling need for further studies of the acute and long-term consequences of energy drinks, with emphasis on contextual use, namely in the setting of physical activity, mental stress, competitive sports and use of other stimulants. There is also need for consideration of greater regulatory oversight of the content and consumption of energy drinks, as well as warning labels, particularly with regard to the risks of consuming multiple drinks over short time periods.

## Figures and Tables

**Table 1:** Comparison of composition and ingredients in several popular energy drinks.

	Bang 16 oz	Five-Hour Energy Extra Strength 1.93 oz	Monster 16 oz	Red Bull 16 oz	Rockstar 16oz
Caffeine	300 mg	230 mg	160 mg	151 mg	160 mg
Calories	0	4	230	220	260
Sugar	0	0	54 g	52 g	62 g
Sodium	40 mg	15 mg	370 mg	200 mg	70 mg
Taurine	Not listed as being present	Listed as part of 2000 mg energy blend	2000 mg	2000 mg	2000 mg
L-Carnitine	Not listed but contains Branched Chain Amino Acid Blend	Not listed as being present	Listed as ingredient in 25,000 mg proprietary energy blend	Listed as ingredient	50 mg
Guarana	Not listed as being present	Not listed as being present	Guarana seed extract listed as ingredient in 25,000 mg proprietary energy blend	Not listed as being present	Guarana seed extract 200 mg
Ginseng	Not listed as being present	Not listed as being present	400 mg of Panax Ginseng	Not listed as being present	Listed in ingredients

All information gathered from product labels and manufacturer websites.
